# Investigating Energetic X-Shaped Flares on the Outskirts of A Solar Active Region

**DOI:** 10.1038/srep34021

**Published:** 2016-09-28

**Authors:** Rui Liu, Jun Chen, Yuming Wang, Kai Liu

**Affiliations:** 1 CAS Key Laboratory of Geospace Environment, Department of Geophysics and Planetary Sciences, University of Science and Technology of China, Hefei 230026, China; 2 Collaborative Innovation Center of Astronautical Science and Technology, Hefei 230026, China; 3 Synergetic Innovation Center of Quantum Information & Quantum Physics, University of Science and Technology of China, Hefei 230026, China; 4 Mengcheng National Geophysical Observatory, School of Earth and Space Sciences, University of Science and Technology of China, Hefei 230026, China

## Abstract

Typical solar flares display two quasi-parallel, bright ribbons on the chromosphere. In between is the polarity inversion line (PIL) separating concentrated magnetic fluxes of opposite polarity in active regions (ARs). Intriguingly a series of flares exhibiting X-shaped ribbons occurred at the similar location on the outskirts of NOAA AR 11967, where magnetic fluxes were scattered, yet three of them were alarmingly energetic. The X shape, whose center coincided with hard X-ray emission, was similar in UV/EUV, which cannot be accommodated in the standard flare model. Mapping out magnetic connectivities in potential fields, we found that the X morphology was dictated by the intersection of two quasi-separatrix layers, i.e., a hyperbolic flux tube (HFT), within which a separator connecting a double null was embedded. This topology was not purely local but regulated by fluxes and flows over the whole AR. The nonlinear force-free field model suggested the formation of a current layer at the HFT, where the current dissipation can be mapped to the X-shaped ribbons via field-aligned heat conduction. These results highlight the critical role of HFTs in 3D magnetic reconnection and have important implications for astrophysical and laboratory plasmas.

Solar flares are the most powerful events in the solar system. A large flare can release ~10^32^ erg of energy (the equivalent of over 20 million 100-megaton hydrogen bombs) in tens of minutes, emitting radiation across the entire electromagnetic spectrum and ejecting relativistic particles into interplanetary space, which may jeopardize space-borne and ground-based technological systems^1^. It is well known that such an energy is gradually accumulated in the corona via emergence of magnetic flux through the photosphere and/or small- and large-scale convective motions in the photospheric layers that shuffle around the footpoints of coronal magnetic field lines^2^. These processes feed magnetic energy and helicity into the solar atmosphere, the latter of which measures the twist and linkage of magnetic field lines^3^. There is mounting observational evidence supporting that magnetic reconnection is the key process in the sudden conversion of the accumulated magnetic free energy into thermal and kinetic energies^4^. In particular, the morphology and dynamics of flare ribbons on the chromosphere, which are produced by field-aligned accelerated electrons and heat conduction, provide valuable diagnosis on the reconnection process in the corona^5^.

Magnetic reconnection tends to occur at the so-called *structural skeletons*^6^,^7^ of magnetic field. Topological skeletons^8^, where the field-line mapping from one footpoint to another is discontinuous, include null points, where the magnetic field vanishes, and separatrix surfaces, which define the boundary of topologically distinct domains. Quasi-skeletons, where the field-line mapping has a steep yet finite gradient, are also known as quasi-separatrix layers (QSLs)^9^. With magnetic connectivity mapped out by the squashing factor *Q*, QSLs can be defined as high-Q structures, whereas *Q* → ∞ at topological skeletons^10^. This indicates a close relation between QSLs and topological skeletons, and a conversion between them under certain conditions is possible^11^. Two separatrix surfaces or QSLs may intersect at a separator or a quasi-separator, respectively. A combination of two intersecting QSLs is termed as a hyperbolic flux tube (HFT)^10^, because of its X-type cross section. Nulls, separators and HFTs are considered to be preferential locations for the concentration of strong currents and the subsequent rapid dissipation^12^,^13^,^14^. Structural skeletons provide a robust and powerful tool to understand complex observations, and also a solid base for the investigation on dynamics and energetics^8^,^15^,^16^.

Major flares almost always occur in the neighborhood of PILs separating strong magnetic fields of opposite polarity in ARs. Only a small fraction of flares occur in plages with only small or no sunspot, which are often associated with filament eruptions^17^,^18^,^19^,^20^,^21^ and hence fall into the category of the classical “two-ribbon” flares, with only a few exceptions in the literature^22^. Typically the two ribbons are connected by flare loops across the PIL, and move away from each other as the flare progresses. This can be well explained by the “standard” flare model^23^,^24^,^25^,^26^. In this two-dimensional model, a rising magnetic flux rope above the PIL stretches the overlying field lines, resulting in a vertical current sheet underneath the rope, where magnetic reconnection occurs successively at an increasingly higher altitude, as a positive feedback is established between the reconnection and the rising of the rope. The two parallel ribbons hence correspond to the footpoints of newly reconnected field lines, because of the assumed symmetry along the PIL.

Recently, flares exhibiting a circular ribbon have received a lot of attention^27^,^28^,^29^,^30^,^31^,^32^,^33^. These flares cannot be accommodated by the classical two-dimensional model, but can well fit into the fan-spine topology of a three-dimensional null point. The circular ribbon is interpreted as the intersection of a dome-shaped separatrix surface, known as the fan surface of the null, with the chromosphere, while the spine field line threading the fan at the null may anchor at a central ribbon inside the circular ribbon and a remote ribbon outside^27^.

Here we present the rare observations of a series of flares exhibiting X-shaped ribbons (also referred to hereafter as X-shaped flares). These flares did not occur in the neighborhood of internal PILs, but repeatedly in the same place on the outskirts of the active region. However, three of them were alarmingly energetic, ranked as M class. These observations demonstrate that even the outskirts of solar active regions has the potential to produce high-level space weather events, and the X-shaped ribbons highlight the three-dimensional nature of magnetic reconnection in flares. As the flares occurred not far away from the solar disk center, the magnetic field measurements were reliable, providing a precious opportunity to study the magnetic topology as well as the evolution, including creation and destruction, of the relevant structural skeletons.

## Results

### Instruments and Datasets

The flares with X-shaped ribbons occurred in NOAA AR 11967, which was monitored by vector magnetograms obtained by the Helioseismic and Magnetic Imager (HMI^34^) onboard the Solar Dynamics Observatory (SDO^35^). The vector magnetograms used in this study were disambiguated and deprojected to the heliographic coordinates with a Lambert (cylindrical equal area; CEA) projection method, resulting in a pixel scale of 0.03° (or 0.36 Mm)^36^. Note that the Carrington system was adopted so that the longitude of a solar feature remains approximately constant.

**Figure 1:**
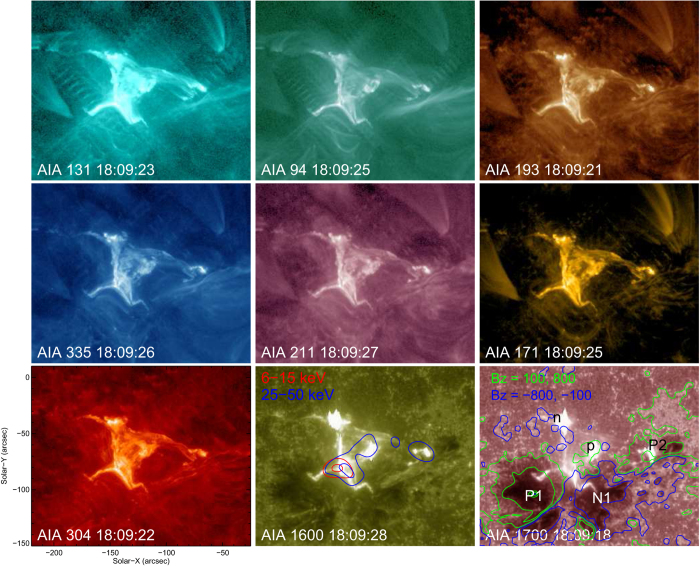
Snapshots of the X-shaped M3.1 flare at 18:11 UT on 2014 February 2 in AIA’s nine UV/EUV passbands. Heliocentric-cartesian coordinates are adopted so that *X* (*Y*) increases towards the solar west (north). The AIA 1600 Å image is superimposed by RHESSI HXR contours at 6–12 (red) and 25–50 (blue) keV, with the contour levels set at 50 and 90 percent of the maximum intensity. The AIA 1700 Å image is superimposed by the contours of the line-of-sight component of the photospheric magnetic field. The green and blue colors indicate positive and negative polarities, respectively, with the contour levels set at ±100 and ±800 Gauss. An animation of AIA images is provided in Supplementary Movie 1.

The flares produced by AR 11967 were observed with the Atmospheric Imaging Assembly (AIA^37^) onboard SDO. AIA includes seven EUV passbands, i.e., 131 Å (primarily Fe XXI for flare plasma, peak response temperature log *T* = 7.05; primarily Fe VIII for ARs, log *T* = 5.6^38^), 94 Å (Fe XVIII, log *T* = 6.85), 335 Å (Fe XVI, log *T* = 6.45), 211 Å (Fe XVI, log *T* = 6.3), 193 Å (Fe XXVI for flare plasma, log *T* = 7.25; Fe XII for ARs, log *T* = 6.2), 171 Å (Fe IX, log *T* = 5.85), 304 Å (He II, log *T* = 4.7), and two UV passbands, i.e., 1600 Å (C IV, log *T* = 5.0) and 1700 Å (continuum). The instrument takes full-disk images with a spatial scale of 0.6 arcsec pixel^−1^ and a cadence of 12 s for EUV and 24 s for UV passbands. Two of the flares (Fig. 1 and Supplementary Figure 1) were also observed in hard X-rays (HXRs) by the Reuven Ramaty High-Energy Solar Spectroscopic Imager (RHESSI^39^).

It is well known that flare emission in 1600 Å forms the flare ribbons, or the feet of flare loops, since this passband is dominated by C VI line emission, an optically thin line formed at 10^5^ K in the upper chromosphere and transition region. The 1700 Å passband is dominated by UV continuum emission formed at the temperature minimum region (4400–4700 K). Flare enhancement in this passband reflects also footpoint emission, typically attributed to photoionization excited by UV emission from above^40^,^41^.

### Flare Observation

**Figure 2:**
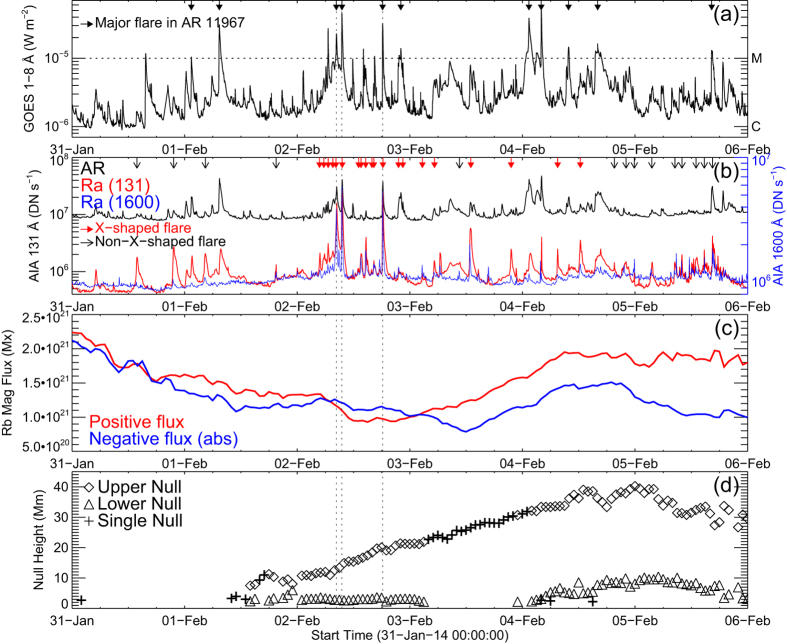
Flares in relation to AR 11967. (**a**) GOES 1–8 Å light curve. Arrows on the top mark the peak times of major flares (M-class and above) in this AR from the beginning of January 31 till end of February 5, during which the AR crossed the solar disk from about 45°E to 45° W. (**b**) Integrated emission over the whole AR in 131 Å (black) and the regional emission integrated over Ra (rectangle in Fig. 3(b)) in 131 Å (red) and 1600 Å (blue). X-shaped flares are marked by red arrows while non-X-shaped flares by black arrows. (**c**) Magnetic fluxes in Rb (Fig. 3(b)). Positive fluxes and absolute values of negative fluxes are denoted in red and blue, respectively. (**d**) Heights of the detected magnetic null points. The lower null is marked with a triangle and the upper null with a diamond when a double null is present. A single null is marked by a ‘+’ symbol. The peak times of the three XMFs are denoted by vertical dotted lines.

**Figure 3:**
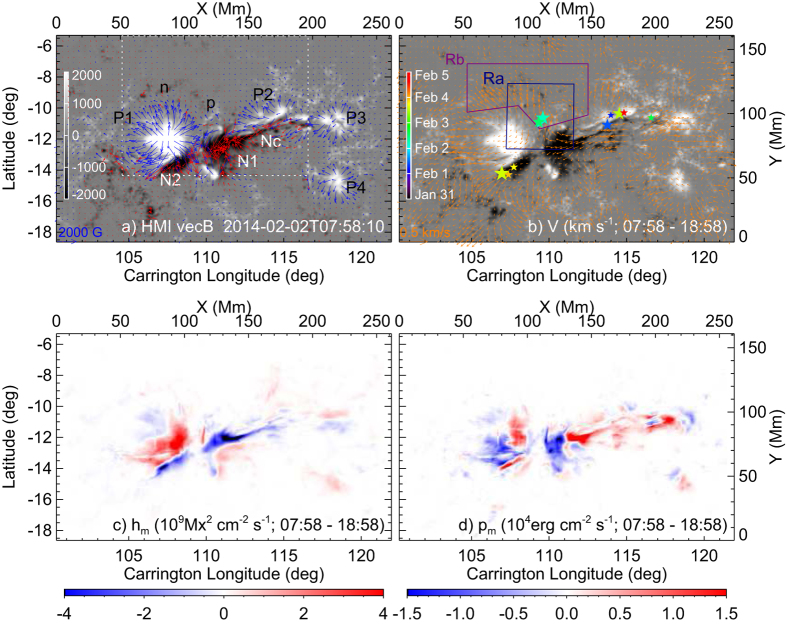
HMI observation of AR 11967. (**a**) Vector magnetogram of AR 11967 immediately before the occurrence of the first XMF at 08:20 UT on 2014 February 2. White and black colors refer to positive and negative *B*_*z*_, respectively, which are scaled to ±2000 G (see the color bar). Red (blue) arrows represent the tangential field components that originate from negative (positive) *B*_*z*_ elements. (**b**–**d**) Maps of photospheric flows, helicity and Poynting flux density averaged over a time period of 11 hrs covering the three XMFs. In Panel (**b**) the locations of all the major flares from January 31 to February 5, as given in Table 1, are marked with star symbols. The colors and sizes of the symbols indicate different times (see the color bar) and flare magnitudes, respectively. The largest size corresponds to the M5.2 flare at 03:57 UT on 2014 February 4. The evolution of the vector magnetic field, flow field, helicity and Poynting flux density is provided in Supplementary Movie 2.

Three flares on 2014 February 2 (Fig. 1, Supplementary Figures 1 and 2) were outliers among the 11 major flares (Table 1; M-class and above; marked by arrows in Fig. 2(a)) that occurred in AR 11967 from the beginning of 2014 January 31 till the end of February 5, when the AR crossed the solar disk from about 45°E to 45° W. These three outliers (peak times marked by vertical lines in Fig. 2) had unique X-shape ribbons and occurred in a facular (or plage) region of AR 11967, where the magnetic field is typically weaker (~100 G), concentrated in much smaller bundles (~1′′) than in sunspots (~1000 G; ~100′′), while all the other non-X-shaped major flares took place near the internal PILs separating sunspots of opposite polarity (cf. Fig. 3(b)). Here the X-shaped major flare (XMF) occurring at 18:11 UT (soft X-ray peak) is taken as an exemplary event (Fig. 1; Supplementary Movie 1), as the other two XMFs taking place at 08:20 and 09:31 UT exhibited almost the same features (Supplementary Figures 1 and 2 and Supplementary Movies 6 and 7). Besides the three XMFs, there were many other weaker flaring activities in this region, as shown by the time series of 131 and 1600 Å emission (Fig. 2(b)) integrated over a rectangular region Ra (Fig. 3(b)) centered on the XMFs, using co-registered AIA images at 5-min cadence, as compared to the integrated 131 Å emission over the whole AR. Most of the X-shaped flares in Ra (marked by red solid arrows at the top of Fig. 2(b)) occurred on February 2 and early February 3, while most of non-X-shaped flares in Ra (black hollow arrows) happened before February 2 and after late February 4. One can also see that some of the non-X-shaped flares did not cause significant response in 1600 Å, while for almost all the X-shaped flares the 131 Å and 1600 Å emission in Ra were highly correlated (Fig. 2(b)). There were even weaker activities missing in this approach, such as the event reported by Yang *et al*.^42^, which exhibited an X morphology in both H*α* and EUV and was interpreted in terms of the classic X-type reconnection. All of these X-shaped flares or flaring activities were confined, without being associated with a coronal mass ejection (CME) or a jet.

It is extraordinary that the flare emission during the impulsive phase had a similar X shape in AIA’s all 9 UV/EUV passbands (Fig. 1). Usually the six EUV passbands excluding 304 Å feature flare loops in the corona, whereas 304, 1600 and 1700 Å passbands feature flare ribbons in the chromosphere. The observed X-shaped emission in EUV, however, must originate from the feet of the flare loops due to the similarity with UV ribbons. It was recently noticed that plasma in flare ribbons can be heated to ~10 MK during the early impulsive phase before flare loops are filled with evaporated chrompospheric plasma, which indicates intense heating of the lower atmosphere^43^,^44^,^45^. The heating mechanism, however, is still under debate. In contrast to UV/EUV emissions, the HXR emission is concentrated at the center of the X shape (Fig. 1), for both the thermal (6–15) and nonthermal (25–50 keV) energy ranges (see Supplementary Figure 3), indicating the projected location where accelerated electrons were interacting with plasma. Two weak, compact nonthermal HXR sources, as compared to the major source at the center of the X shape, were co-located with the most intense UV/EUV patches along the northwestern arm of the X shape. It is clear that this X morphology was not produced by a single classic X-type magnetic reconnection, or reconnections between two crossing, current-carrying loops^46^, either of which would have produced four flare kernels in UV and/or HXRs. Instead, we observed extended X-shaped flare ribbons in UV/EUV and a compact HXR source at the center of the X shape. The analysis of photospheric magnetic fields will shed light on the nature of the reconnection.

Superimposing the contours of the line-of-sight (LOS) component of the photospheric magnetic field onto the UV 1700 Å image, one can see that the adjacent endpoints of the X shape were associated with opposite polarities (Fig. 1; Supplementary Figures 1 and 2). These four flux concentrations are labeled P1-N1 and p-n. P1 and N1 were associated with two sunspots, whereas p and n with sporadic patches of weak field in the facular region (see also Fig. 3(a)). A third sunspot (labeled P2) was also involved, with the northwestern “arm” of the X shape extending to the immediate neighborhood of P2.

### Evolution of Active Region

Immediately prior to the three XMFs (07:58 UT), the AR was characterized by six major sunspots (Fig. 3(a)), four of them were associated with positive polarity (labeled P1–P4) and two (labeled N1 and N2) with negative polarity. P2 and P4 decayed and became diffused by February 5, while other sunspots were relatively stable. Figure 3(b–d) show the maps of photospheric flows [km s^−1^], helicity flux density *h*_*m*_ [Mx^2^ cm^−2^ s^−1^] and Poynting flux density *p*_*m*_ [erg cm^−2^ s^−1^], which are averaged over a time interval of about 11 hrs covering the XMFs (see Methods). These maps reveal a persistent evolutionary pattern starting in the late January 31 until the mid February 4 (see Supplementary Movie 2), viz., a significant flux of negative polarity emerged to the east of P3, and then migrated eastward into N1 (Fig. 3(b)), forming a channel of negative magnetic fluxes (labeled Nc in Fig. 3(a)) and also a channel of intense negative *h*_*m*_ (Fig. 3(c)) and positive *p*_*m*_ (Fig. 3(d)) between N1 and P3 and to the south of P2. As they approached P1, the flows bifurcated, some were diverted northward, integrated with the moat flows^47^ of P1 and N1, into the facular region where the three XMFs occurred (olive star symbols in Fig. 3(b)), and others were diverted southeastward along the PIL between P1 and N2. Of the 8 non-X-shaped major flares (see also Table 1), 5 occurred along Nc and 3 along the PIL between P1 and N2.

It is also remarkable that there was significant flux cancellation ongoing in the facular region to the north of P1-N1, which might be driven by the moat flows of P1 and N1 (see Supplementary Movie 2). Positive and negative fluxes integrated in an irregular region Rb (Fig. 3(b)) are shown in Fig. 2(c). Rb was so selected as to avoid the sunspots P1, N1, and P2. The cancellation started from the beginning of January 31 until mid February 3. After that it appeared to be dominated by the emergence of positive fluxes in Rb and the migration of negative fluxes from N1 into Rb with these flux elements being detached from N1. Studies have argued for the association between flux cancellation and magnetic reconnection^48^.

### Magnetic Topology

To understand the magnetic connectivities in AR 11967, we performed the extrapolation of the coronal potential field on an hourly cadence from January 31 to February 5 (see Methods). Potential field maintains the basic topology although it can only be regarded as a zero-order approximation of the real coronal field. The robustness of structural skeletons has been demonstrated by earlier studies employing various coronal field models^15^,^16^,^49^, and also in the present study by switching on and off a “pre-processing” procedure on the photospheric boundary (see Methods). However, current-carrying structures, e.g., twisted flux tubes, cannot be recovered in the potential field, and the assumption adopted by the null-locating algorithm^50^,^51^ (see also Methods), that the magnetic field approaches zero linearly toward nulls, might break down with the presence of strong currents.

**Figure 4:**
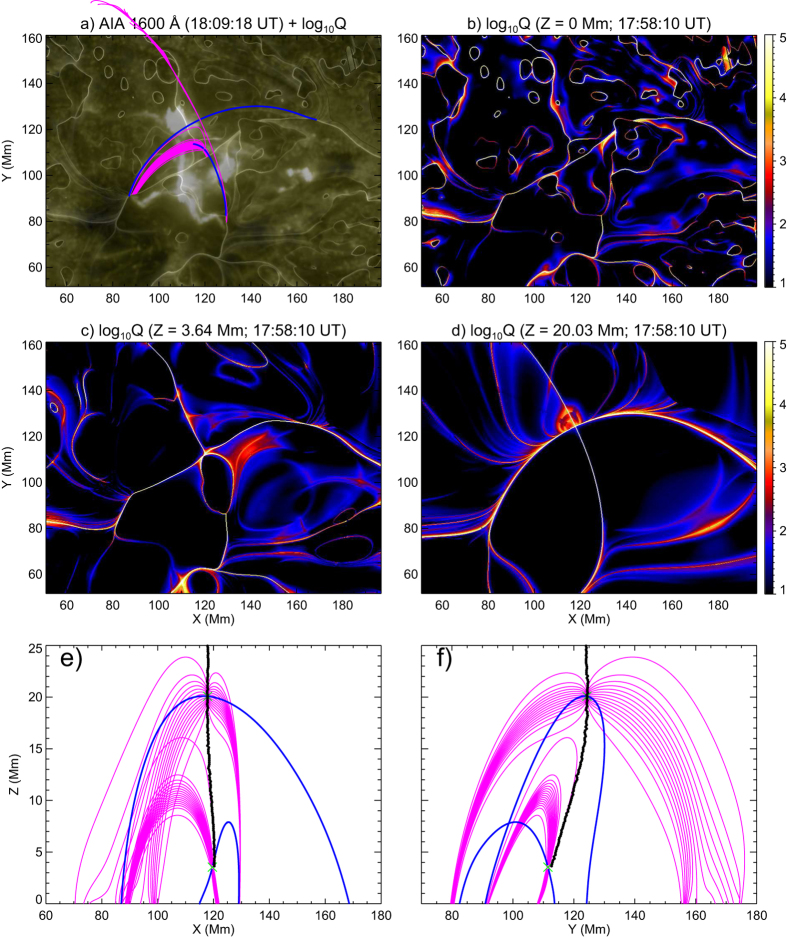
Magnetic topology prior to the XMF at 18:11 UT on 2014 February 2. (**a**) A UV 1600 Å image taken during the impulsive phase of the flare (same as in Figure 1) and remapped with the CEA projection is blended with the log *Q* map on the photosphere in Panel (**b**), which is calculated with the HMI magnetogram at 17:58 UT. Panels (**c**,**d**) show two cuts of log *Q* at different heights just above the lower null and below the upper null, respectively. Field lines traced in the neighborhood of the double null, with fan (spine) field lines in magenta (blue), are projected in X-Y (Panel a), X-Z (Panel e), and Y-Z (Panel f) planes. The black dots in (**e**,**f**) indicate the visually determined center of the X shape in log *Q* at different heights. The X shape is complicated below the lower null, hence its central position is not shown here. An animation of log *Q* at different heights is provided in Supplementary Movie 3.

For each potential field, we sought for magnetic null points within a box volume, whose bottom was the rectangular region indicated in Fig. 3(a). Most of the identified nulls were located in the facular region to the north of P1 and N1 (see Methods and Supplementary Figures 4 and 5), and sometimes an interesting double-coronal-null configuration was present, i.e., two nulls were separated in height but have similar X-Y positions. One can see from Fig. 2(d) that double nulls started to appear in late February 1, disappeared in early February 3, and reappeared in the middle of February 4. It is noteworthy that most of the X-shaped flares occurred when the double-null configuration was present and when there was significant flux cancellation in the facular region of interest (Fig. 2). Field lines traced in the neighborhood of the double null just prior to the XMF at 18:11 UT on 2014 February 2 are shown in Fig. 4(a,e,f) with fan (spine) field lines in magenta (blue). The upper null was located at *Z* = 20.08 Mm and the lower null at *Z* = 3.55 Mm (marked by green crosses in Fig. 4(e,f)).

**Figure 5:**
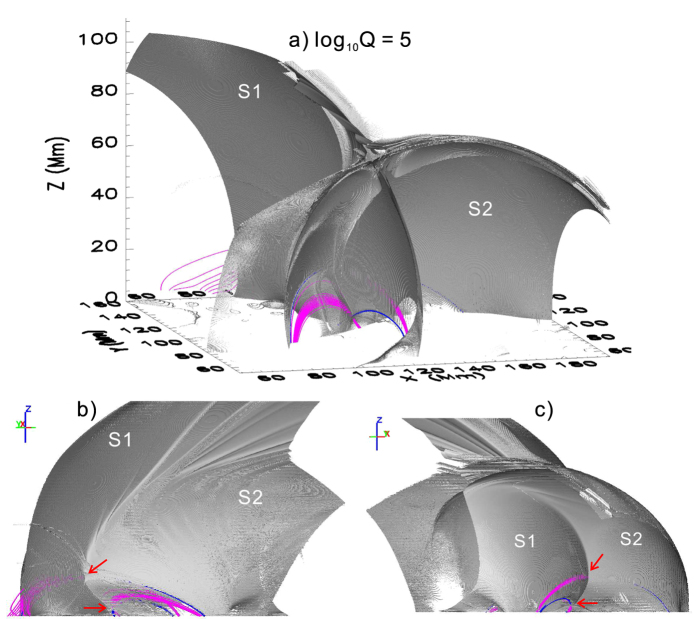
QSLs prior to the XMF at 18:11 UT on 2014 February 2. (**a**) Isosurfaces of log *Q* = 5, superimposed by field lines traced in the neighbourhood of the double null, with fan (spine) field lines in magenta (blue). Bottom panels show two side views of the isosurfaces from the east (**b**) and the southwest (**c**), respectively. The null locations are roughly indicated by red arrows. A 360 deg side view of the isosurfaces is provided in Supplementary Movie 4.

We also calculated the squashing factor *Q* in the same box volume (see Methods). The isosurfaces of log_10_*Q* = 5 (Fig. 5) consist of two major intersecting QSLs, labeled ‘S1’ and ‘S2’, whose footprints on the photosphere correspond to the high-*Q* lines in Fig. 4(a,b). The intersection of S1 and S2 is a quasi-separator by definition. It is well defined immediately above the lower null, as demonstrated by the X-shaped morphology in horizontal cutting planes of log_10_*Q* (Fig. 4(c,d); see also Supplementary Movie 3), the characteristic of an HFT. The center of X was visually determined and plotted as black dots in Fig. 4(e,f), showing that the two nulls are located along the quasi-separator. Viewing the QSLs from different perspectives (Fig. 5 and Supplementary Movie 4), one can see that the fan (spine) field lines of the upper null, partially visible through the isosurfaces, are embedded within S1 (S2), while the reverse is true for the lower null. The fan plane of the upper null is hence bounded by the spine of the lower null from below within S1, while the fan plane of the lower null is bounded by the spine of the upper null from above within S2. The intersection of the two fan planes hence determines a separator connecting the two nulls^52^, which in our case is embedded within the quasi-separator. The quasi-separator extends beyond the upper null, up to as high as about 60 Mm, inclining southward above the upper null and northward below it (Fig. 5(b,c) and Supplementary Movie 4). With or without the nulls, the overall configuration of the HFT remained almost the same as in Fig. 5 (see also Supplementary Figure 6) during the time period investigated, due presumably to the relatively slow evolution of AR 11967 (see Supplementary Movie 2). Apparently, the recurrence of X-shaped flares in this region owed its origin to the relatively stable presence of this HFT.

The double null’s fans and spines yield an X shape, but its mismatch with the flare ribbons is not negligible (Fig. 4(a)). In particular, the southwestern arm of the X shape is left unaccounted for, as the upper null’s fan is bounded by the lower null’s spine from below. Hence, the double null and the separator cannot be solely responsible for the observed flare ribbons. On the other hand, it was found that the X-shaped ribbons are better matched by high-Q lines on the photosphere (Fig. 4(a); see also Methods), which delineate the footprints of S1 and S2. The flare ribbons are hence suggested to be mainly energized by magnetic reconnections at the HFT.

**Figure 6:**
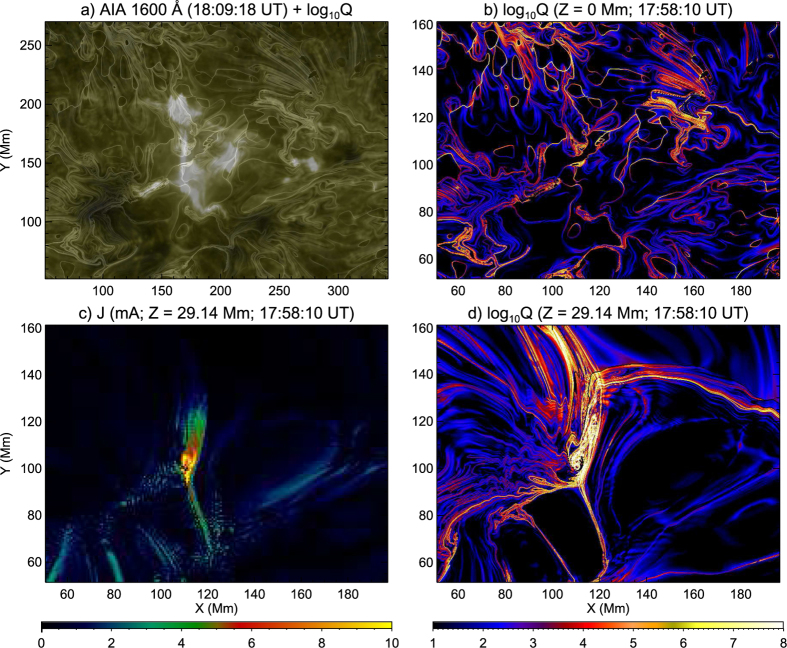
Magnetic topology as revealed by a NLFFF model for the XMF at 18:11 UT on 2014 February 2. Panel (a) shows a blend of UV 1600 Å image and the photospheric log *Q* map, the latter of which is shown in Panel (b). Panels (c) and (d) show a horizontal cut of the electric current and log *Q* at *Z* = 29 Mm, respectively. An animation of log *Q* at different heights is provided in Supplementary Movie 5.

However, to produce the flare, the configuration must carry a substantial electric current. We built a nonlinear force-free field (NLFFF) with the “weighted optimization” method to model the coronal field^2^,^53^. The vector magnetogram at 17:58 UT was “pre-processed”^54^ to minimize the net force and torque in the observed photospheric field before being taken as the boundary. By obtaining the squashing factor *Q* for this NLFFF in the same box volume as above, we found that the basic topology can still be interpreted as two intersecting QSLs, yet the intersection was no longer a 3D curve as in the potential field, but a very complex structure (Fig. 6(d) and Supplementary Movie 5), with the concentration of electric currents (Fig. 6(c)), yet with no null being identified numerically. However, the overall morphology of the intersection retains the X shape, the basic characteristic of an HFT. We hence determined that the HFT in the NLFFF is equivalent in topology to, but much more complex in geometry than, its counterpart in the potential field. Its footprints at the photosphere also compare favorably with the X-shaped flare ribbons (Fig. 6(a)), which strengthens our conclusion that the flare ribbons are closely associated with the HFT. Similar results were found for the other two XMFs.

## Discussion

A comparison with an idealized quadrupole field consisting of two oppositely directed dipoles with different magnitudes (see Methods) may shed light on the observed topology, which appeared to involve mostly a local quadrupole field as represented by P1-N1 and p-n. An HFT inclining towards the weaker dipole was identified at the intersection of two high-Q surfaces in this idealized quadrupole field (Supplementary Figures 7, 8 and 9). When the two dipoles are strictly anti-parallel, a null line was found to be coincident with the HFT. Each point along the null line is associated with two spines, whose footpoints collectively match the X-shaped footprint of the HFT. The HFT persists while the null line perishes when the two dipoles are not perfectly anti-parallel. However, the arms of this X shape diverge toward the stronger dipole and converge toward the weaker one, and the strength of the two dipoles has to be comparable to yield a symmetric X shape. In contrast, the observed flare ribbons were more extended at the weak field side p-n than at the strong side P1-N1 (Fig. 1 and Supplementary Figures 1 and 2). The separator in observation can be regarded as a generalized form of the null line and did incline toward the weak field as the null line, but above the upper null, the quasi-separator inclined backwards toward the strong field. This suggests that the production of the X-shaped flare ribbons was not just dictated by the local quadrupole field, but must be regulated by all the other flux concentrations as well as photospheric flows within the AR.

The above analysis converges to a self-consistent physical scenario: the flux emergence and flows associated with the channel Nc energize the AR by injecting magnetic helicity and Poynting fluxes into the solar atmosphere, which results in the accumulation of magnetic free energy and stress in the neighborhood of the internal PILs, as well as in the facular region of interest, above which the HFT is probably pinched into a current layer, as suggested by the NLFFF model as well as studies with a broad variety of photospheric motions applied to its feet^12^,^13^,^14^. When magnetic reconnections occur at this current layer, the field-aligned heat conduction channels out the energy released at the HFT and produces the UV/EUV flare ribbons residing at the high Q-lines that delineate the HFT footprints. In contrast, the HXR emission was concentrated at the center of the X shape, which suggests that most of the HXR-producing electrons were thermalized at the HFT, with only a fraction of them leaking out to produce sporadic HXR emission at the flare ribbons (Fig. 1 and Supplementary Figures 1 and 3). How the electrons were accelerated and trapped at the HFT is beyond the scope of this study, but certainly an important area to pursue for future research. The homologousness of these X-shaped flares can be attributed to the repetitive energization and relaxation of the HFT, which proved to be persistent during the time period investigated. These results provide insight into the nature of 3D magnetic reconnection in solar flares, which could be constructive in the efforts of flare forecasting, and may also have important implications for other astrophysical as well as laboratory plasmas.

## Methods

### Helicity and Poynting Flux

The flow field in Fig. 3(b) was obtained by applying the Differential Affine Velocity Estimator for Vector Magnetograms (DAVE4VM)^55^ to the time-series of deprojected, registered vector magnetograms. The window size used in DAVE4VM was chosen to be 19 pixels, following Liu *et al*.^56^.

With the flow field, we were able to calculate the relative helicity flux across its photospheric boundary *S* with the following formula^3^,^56^,^57^:


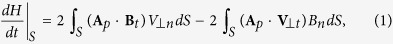


where **A**_*p*_ is the vector potential of the reference potential field **B**_*p*_ that shares the same *B*_*z*_ on the photospheric boundary as the observation; *t* and *n* refer to the tangential and normal directions, respectively. **V**_⊥_ is the photospheric velocity perpendicular to magnetic field lines, which is obtained by subtracting the field-aligned plasma flow (**V** · **B**)**B**/*B*^2^ from the velocity derived by DAVE4VM^57^. The two terms on the right hand side of Eq. 1 describe helicity injection due to flux emergence (*V*_⊥*n*_) and photospheric motions (**V**_⊥*t*_) that shear and braid field lines, respectively. Similarly, both flux emergence and tangential motions contribute to the Poynting flux across the photospheric boundary^58^,


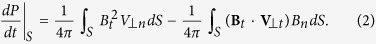


Here our error analysis follows Appendix B in Liu *et al*.^59^. The typical errors of helicity and Poynting fluxes are of order 10^36^ Mx^2^ s^−1^ and 10^25^ erg s^−1^, respectively, less than 1 part in 10. For AR 11967 as a whole (Supplementary Figure 10), we found no significant injection of magnetic flux [Mx], helicity flux [Mx^2^ s^−1^], or Poynting flux [erg s^−1^] during the time period of interest. An injection of helicity and Poynting fluxes started from the early February 5, but apparently it was not related with any major flares. On the other hand, examining the flux density maps in detail (Fig. 3(c,d)), we found a significant local injection of negative helicity flux and positive Poynting flux, associated with the eastward flows in the channel Nc.

### Extrapolation of Coronal Potential Field

We calculated the potential field with both the Fourier transformation^60^ and the Green function method. Both the original and a “pre-processed” *B*_*z*_ was adopted as the photospheric boundary of the extrapolation. The aim of the pre-processing, as applied to the 2 × 2 rebinned Space-Weather HMI Active Region Patches data, was to make the vector magnetograms best suit the force-free condition^54^.

The calculation was carried out within a box of 720 × 344 × 256 uniformly spaced grid points, corresponding to 524.5 × 250.6 × 186.5 Mm, whose photospheric FOV spanned [86.6, 129.8] in Carrington longitudes, and [−23.4, −2.7] in latitudes. Magnetic flux was roughly balanced within the FOV, the ratio between positive and (absolute) negative flux was 1.07 at the beginning of January 31, increasing more or less monotonously toward 1.36 by the end of February 5. The results shown in Figs 4 and 5 were given by the potential field constructed with the Fourier method and the pre-processed boundary. Other potential fields yielded very similar results as far as magnetic topology is concerned (Supplementary Figures 4 and 5).

### Map of Magnetic Connectivities

We investigated magnetic connectivities within the extrapolated field by tracing field lines pointwise with a fourth-order Runge-Kutta method to ensure high precision. The mapped footpoints of field lines were used to calculate the squashing factor *Q* of elemental magnetic flux tubes^10^. Basically, for a mapping through the two footpoints of a field line 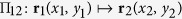, the squashing factor associated with the field line is^10^


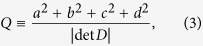


where *a*, *b*, *c*, *d* are elements of the Jacobian matrix


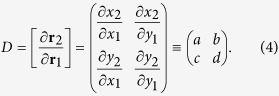


Quasi-separatrix layers (QSLs) as defined by high-*Q* values are often complex three-dimensional structures, hence their visualization can be facilitated by calculating *Q* in a 3D volume box. This was done by stacking up *Q*-maps in uniformly spaced cutting planes^59^. In Fig. 5, the original grids were refined by 4 times in the calculation of *Q*.

### Identification of Null Points

To locate null points in a 3D magnetic field with uniform grids, we assume that within each cell the field is linear or trilinear, following Haynes & Parnell^51^. Under this assumption, a cell is excluded in the further analysis if any of the three magnetic field components [*B*_*x*_, *B*_*y*_, *B*_*z*_] is nonzero and of the same sign at each corner of the cell. More cells can be excluded by checking whether the intersection curves of the isosurfaces *B*_*x*_ = 0, *B*_*y*_ = 0, and *B*_*z*_ = 0 pierce the cell faces in pair^51^. Two methods are used to locate the null position to subgrid precision. One may conduct a brute-force search for min *B* in each candidate cell. First the cell is uniformly divided into (say 1000) miniature cells. The miniature cell centered on the grid point with min *B* is further divided and a new min *B* is located. This is done recursively until the number of recursion exceeds, say, 3 times, or, min *B* < 10^−4^ G. A null point is located if the latter condition is satisfied. Alternatively, one may solve **B** = 0 with the iterative Newton-Raphson method^51^. We confirmed that null points detected in both methods are consistent within the limits of numerical accuracy. However, the Newton-Raphson method is generally more efficient and has the potential to identify multiple nulls within a candidate cell, e.g., in the case of a null line (see below), if one initiates the iteration from a sufficiently large number of subgrid points.

The local magnetic structure in the vicinity of a null point is determined by the tensor 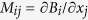
50. The structure is relatively simple for potential fields, since without currents **M** is symmetric and has three real eigenvalues, whose sum is zero. For an isolated null, there is one eigenvalue of opposite sign to the other two, and its associated eigenvector determines the spine while the other two eigenvectors define the fan plane. This differs for a non-isolated null, as exemplified by a null line in an idealized quadrupole field (see below).

There are subtle differences for nulls identified in the potential field constructed with different methods (Green function vs. Fourier transform) and different boundary conditions (original vs. pre-processsed), but the time period when the double-null configuration was present and the locations of these nulls are essentially the same (Supplementary Figures 4 and 5). This demonstrates the robustness of such topological skeletons.

### Idealized Quadrupole Field

Here an idealized quadrupole field, similar to Sun *et al*.^29^, was employed to shed light on the magnetic topology involved in the X-shaped flares. For simplicity, two dipoles were placed in an opposite direction below a Cartesian computation domain 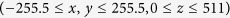: 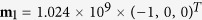 at 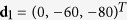, and 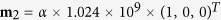 at **d**_2_ = (0, 60, −80)^*T*^, where *α* is a fraction number between 0 and 1. The sum of the two dipole fields yields a potential field with a magnitude of 2000 G at the photosphere (*z* = 0),


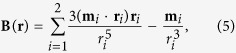


where 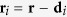. With this setup, **m**_*i*_ · **r**_*i*_ = 0 and *B*_*y*_ = *B*_*z*_ = 0 at the plane *x* = 0. A null line along which **B** = 0 was found to be a circular curve within this plane of symmetry, i.e.,


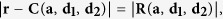


where


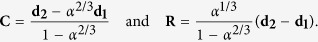


When *α* = 1, however, it degenerates into a line within the same plane,


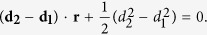


We have tested our null-locating code with this quadrupole field and found that all the nulls identified are located along the analytical null line within the limits of numerical accuracy.

Since **B** is potential, we again have three real eigenvalues for **M** = [∂**B**/∂**r**] given an arbitrary point on the null line, but one of the eigenvalues is precisely zero, say, 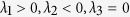, and 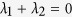. Formally, a position vector **r** on a field line near the null can be written as^50^


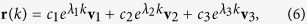


where **v**_*i*_ are eigenvectors, *k* is an arbitrary parameter and *c*_*i*_ are constant along the field line, whose behavior near the null is determined as follows,


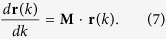


Hence, when one traces a field line forward (*k* → ∞) or backward (*k* → −∞) away from the null, the **v**_3_ term in Eq. 6 becomes irrelevant, and all the field lines are either directed away from the null along **v**_1_ or toward the null along **v**_2_. Thus, each point on the null line owns two spines. Supplementary Figure 7 gives three examples with *α* = [0.2, 0.5, 0.8]. One can see that the null line inclines toward the weaker dipole and that the footpoints of all the spines collectively produce an X shape, if the magnitudes of the two dipoles are comparable.

Mapping out the magnetic connectivities (Supplementary Figure 8), one can see that the null line corresponds to the intersection of two high-Q surfaces, whose footprints are coincident with the footpoints of the spines. This again demonstrates the intimate relation between topological skeletons and QSLs^10^,^11^. However, a slight rotation of **m**_2_ about the *z*-axis breaks the symmetry and the null line disappears completely. Only one single null is identified, when the rotational angle is within about ±20°, beyond which no null is found within the computational domain. In contrast, the HFT structure is stable; it again leans toward the weaker dipole, and its footprint leaves a skewed X shape on the surface when *α* approaches unit (Supplementary Figure 9).

## Additional Information

**How to cite this article**: Liu, R. *et al*. Investigating Energetic X-Shaped Flares on the Outskirts of A Solar Active Region. *Sci. Rep.* **6**, 34021; doi: 10.1038/srep34021 (2016).

## Supplementary Material

Supplementary Information

Supplementary Movie S1

Supplementary Movie S2

Supplementary Movie S3

Supplementary Movie S4

Supplementary Movie S5

Supplementary Movie S6

Supplementary Movie S7

Supplementary Movie S8

Supplementary Movie S9

## Figures and Tables

**Table 1:** List of Major Flares (M-class and above) in AR 11967.

Date	Class	Peak	Carr_Lon	Carr_Lat	Instrument
2014/02/01	M1.0	01:25	113.987	−10.463	AIA
2014/02/01	M3.0	07:23	113.759	−11.086	RHESSI
**2014/02/02**	**M2.2**	**08:20**	**109.400**	**−10.995**	**RHESSI**
**2014/02/02**	**M4.4**	**09:31**	**109.602**	**−10.641**	**AIA**
**2014/02/02**	**M3.1**	**18:11**	**109.325**	**−10.898**	**RHESSI**
2014/02/02	M1.3	22:04	116.584	−10.625	RHESSI
2014/02/04	M3.8	01:23	114.536	−10.355	RHESSI
2014/02/04	M5.2	04:00	106.954	−14.215	AIA
2014/02/04	M1.4	09:49	107.728	−13.806	RHESSI
2014/02/04	M1.5	16:02	107.377	−14.291	RHESSI
2014/02/05	M1.3	16:20	114.830	−10.301	AIA

The Carrington longitudes and latitudes of the flare locations are given by the peak emission loci of RHESSI HXR sources at 6–12 keV or by the peak 131 Å emission loci in the flaring regions when RHESSI data were not available. The three X-shaped flares are shown in boldface. All the events were ‘confined’ without being associated with a coronal mass ejection (CME).
