# Supplementary material for: Investigating Energetic X-Shaped Flares on the Outskirts of A Solar Active Region
Source: Sci Rep. 2016 Sep 28;6:34021. doi: 10.1038/srep34021 (PMC5039731; doi:10.1038/srep34021)
Supplement: Supplementary Information [file srep34021-s1.pdf]

Supplementary Information for "Investigating Energetic X-Shaped Flares on the Outskirts of A Solar Active Region"

Rui Liu[1,2,*], Jun Chen[1], Yuming Wang[1,3], Kai Liu[1,4]

[1]*CAS Key Laboratory of Geospace Environment, Department of Geophysics and Planetary Sciences, University of Science and Technology of China, Hefei 230026, China*

[2]*Collaborative Innovation Center of Astronautical Science and Technology, Hefei 230026, China*

[3]*Synergetic Innovation Center of Quantum Information & Quantum Physics, University of Science and Technology of China, Hefei 230026, China*

[4]*Mengcheng National Geophysical Observatory, School of Earth and Space Sciences, University of Science and Technology of China, Hefei 230026, China*

Supplementary Notes

Supplementary Figures 1 (Supplementary Movie 6) and 2 (Supplementary Movie 7) show the AIA observations of the other two XMFs occurring at 08:20 UT and 09:31 UT on 2014 February 2, respectively. The magnetic topology relevant to the X-shaped ribbons in these two flares was similar to the flare at 18:11 UT (Figures 4–6), with a double null embedded in the intersection of two high-Q surfaces (Supplementary Figure 6 and Supplementary Movies 8 and 9).

The XMFs at 08:20 UT (Supplementary Figure 1) and 18:11 UT (Figure 1) were observed in HXRs by RHESSI. In both flares, we integrated the HXR spectra over 40 s around the peak of the nonthermal HXR bursts (Supplementary Figure 3) and found that they can be well fitted with an exponential thermal function (red) and a nonthermal power-law function (blue).

Supplementary Figures

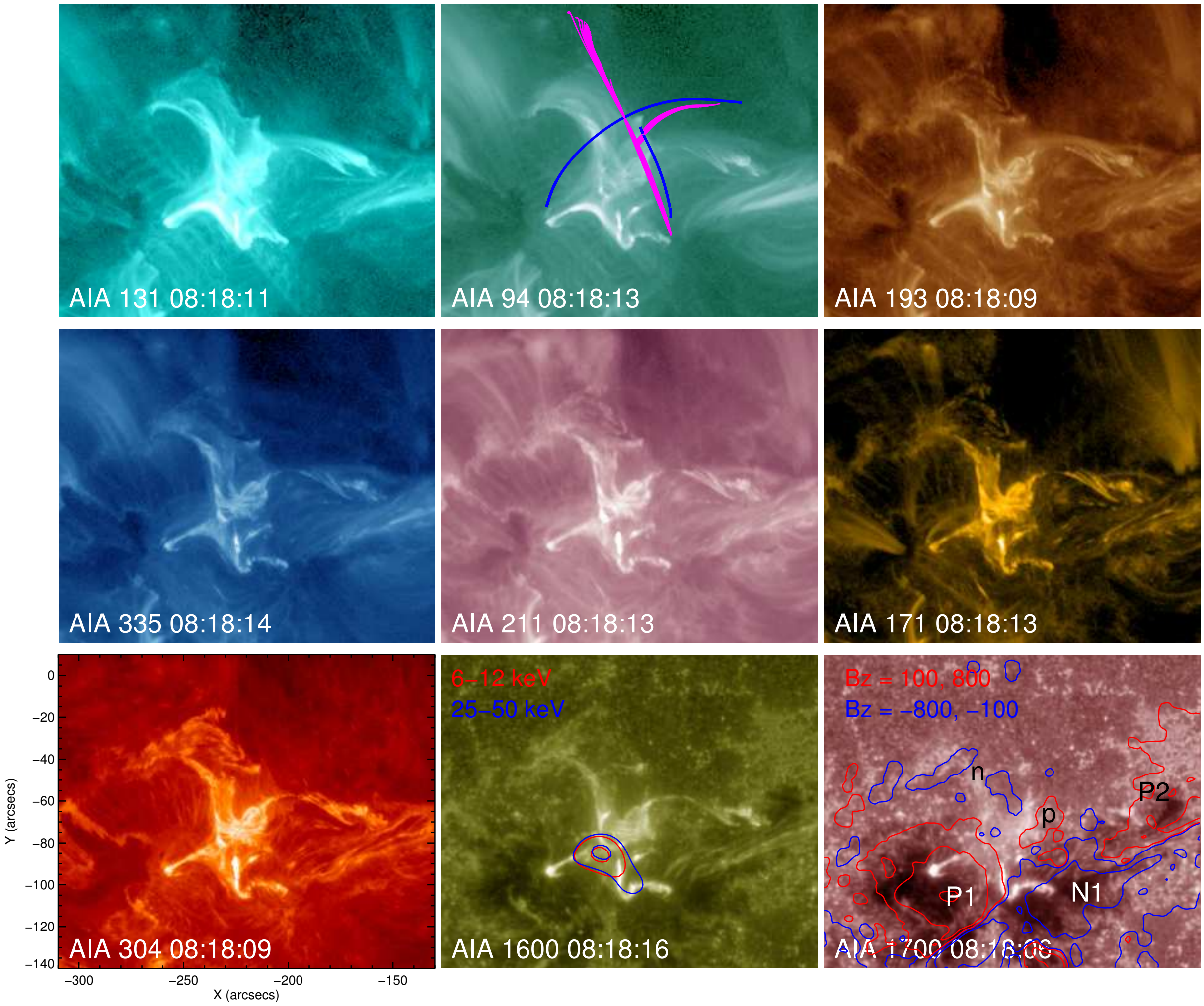

Supp. Fig. 1: A snapshot of the M2.2 flare at 08:20 UT on 2014 Feburary 2 in AIA's nine UV/EUV passbands. The spine (blue) and fan (magenta) field lines of two coronal null points are projected onto the AIA 94 Å image. The AIA 1600 Å image is superimposed by RHESSI HXR contours at 6–12 (red) and 25–50 (blue) keV, with the contour levels set at 50 and 90 percent of the maximum intensity. The AIA 1700 Å image is superimposed by the contours of the LOS component of the photospheric magnetic field. The red and blue colors indicate positive and negative polarities, respectively, with the contour levels set at $\pm$100 and $\pm$800 Gauss. An animation of AIA images is provided in Supplementary Movie 6.

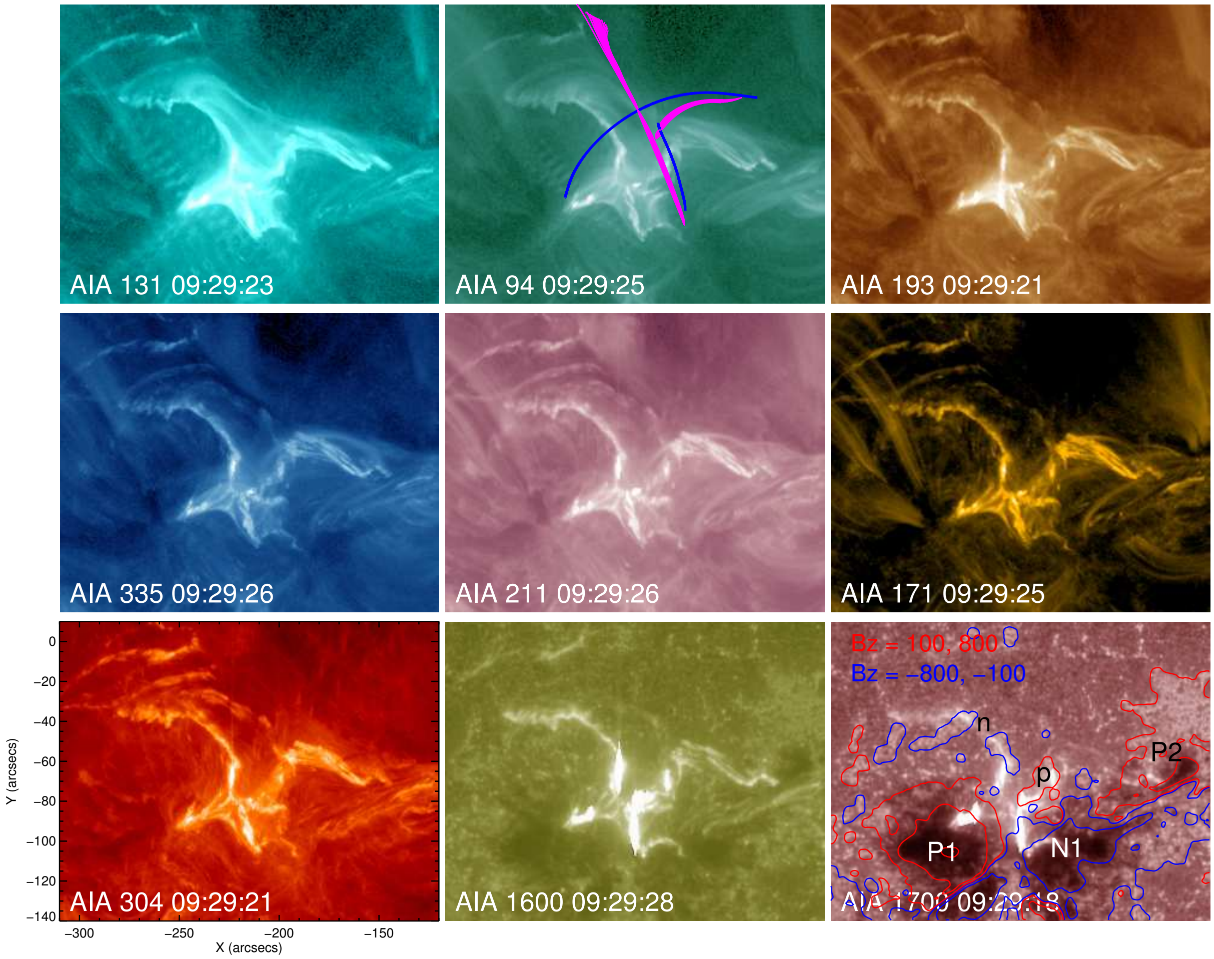

Supp. Fig. 2: A snapshot of the M4.4 flare at 09:31 UT on 2014 Feburary 2 in AIA's nine UV/EUV passbands. The spine (blue) and fan (magenta) field lines of two coronal null points are projected onto the AIA 94 Å image. The AIA 1700 Å image is superimposed by the contours of the LOS component of the photospheric magnetic field. The red and blue colors indicate positive and negative polarities, respectively, with the contour levels set at ±100 and ±800 Gauss. An animation of AIA images is provided in Supplementary Movie 7.

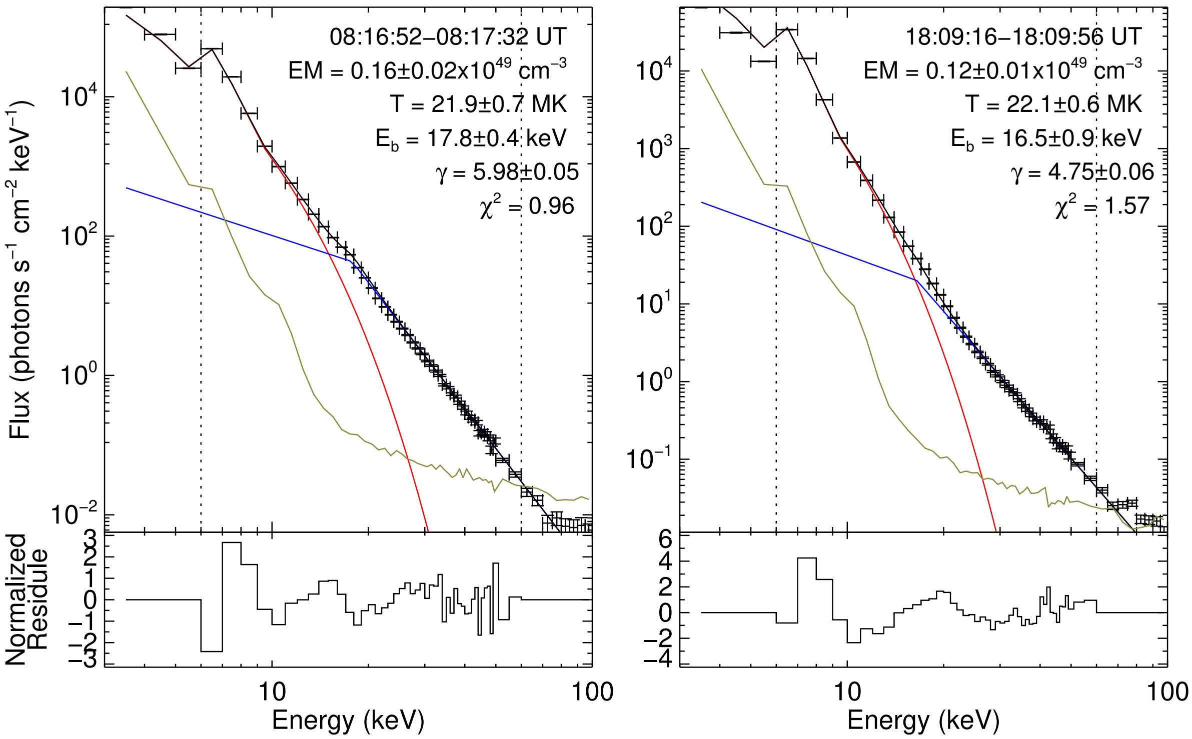

Supp. Fig. 3: Flare spectroscopy for the M2.2 flare at 08:20 UT and M3.1 flare at 18:11 UT on 2014 February 2. RHESSI spectra at the flare peak are fitted in an energy range of 6–100 keV (denoted by dotted lines) with an exponential thermal function (red) and a broken power-law function (blue) with the spectral index γ below the broken energy E_b being fixed at 1.5. The background is shown in gray.

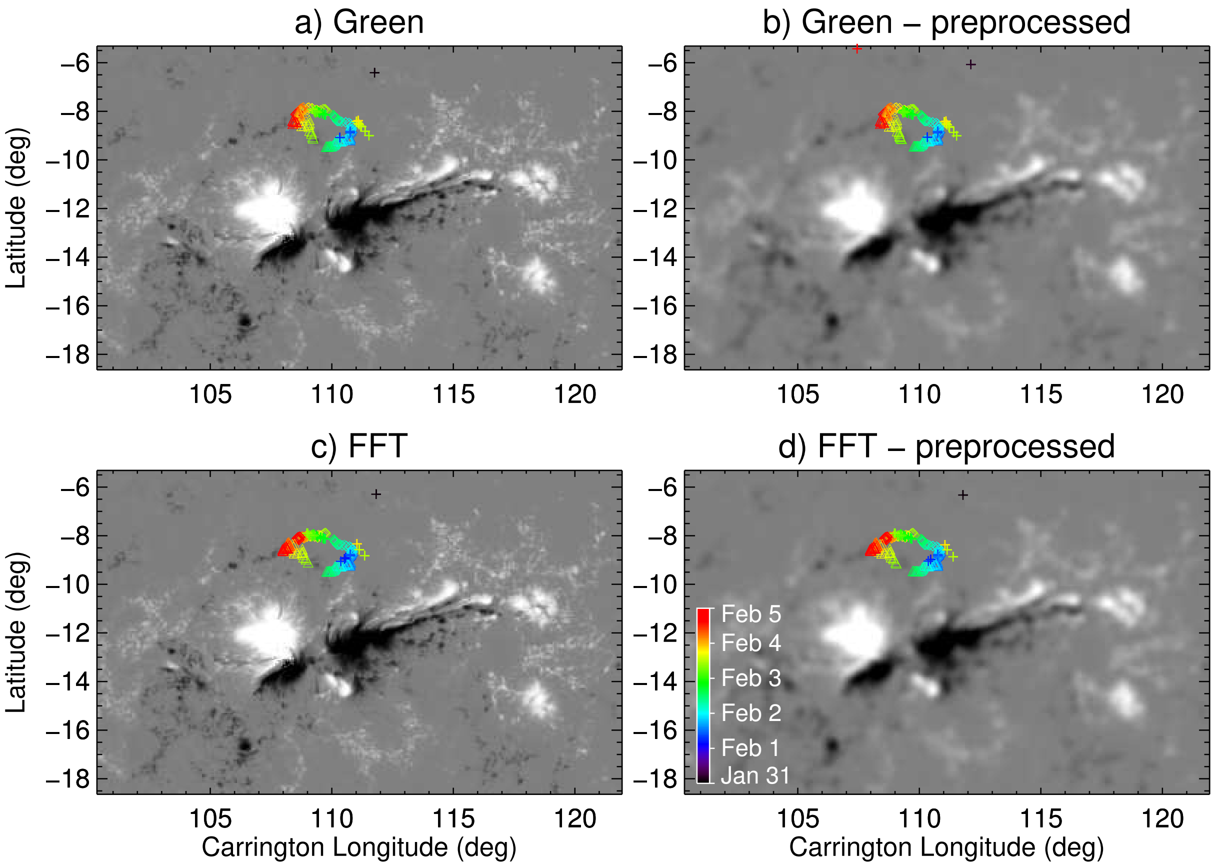

Supp. Fig. 4: Null locations projected on the photospheric boundary used for the potential field extrapolation. The nulls are identified in potential fields constructed with different methods (Green function vs. fast Fourier transform) and different boundary conditions (original vs. pre-processsed). Same as in Figure 2, the lower (upper) null is marked with a triangle (diamond) for double nulls, while a single null is marked by a '+' symbol. The color of symbols indicates the time when the null was present (see the color bar).

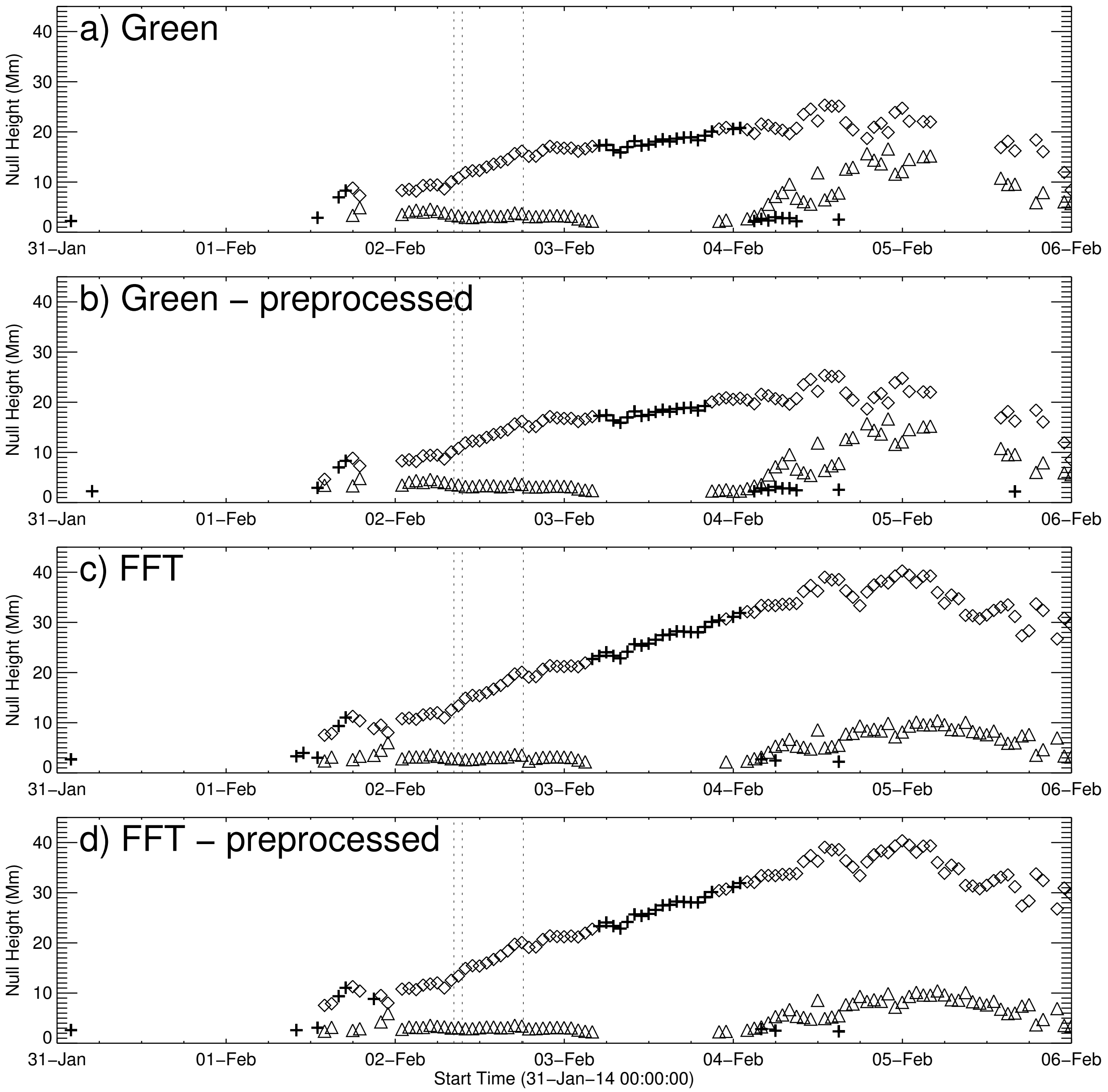

Supp. Fig. 5: Null heights with time. The nulls are identified in potential fields constructed with different methods (Green function vs. fast Fourier transform) and different boundary conditions (original vs. preprocesssed). Vertical dotted lines indicate the peak times of three XMFs. Same as in Figure 2, the lower (upper) null is marked with a triangle (diamond) for double nulls, while a single null is marked by a '+' symbol.

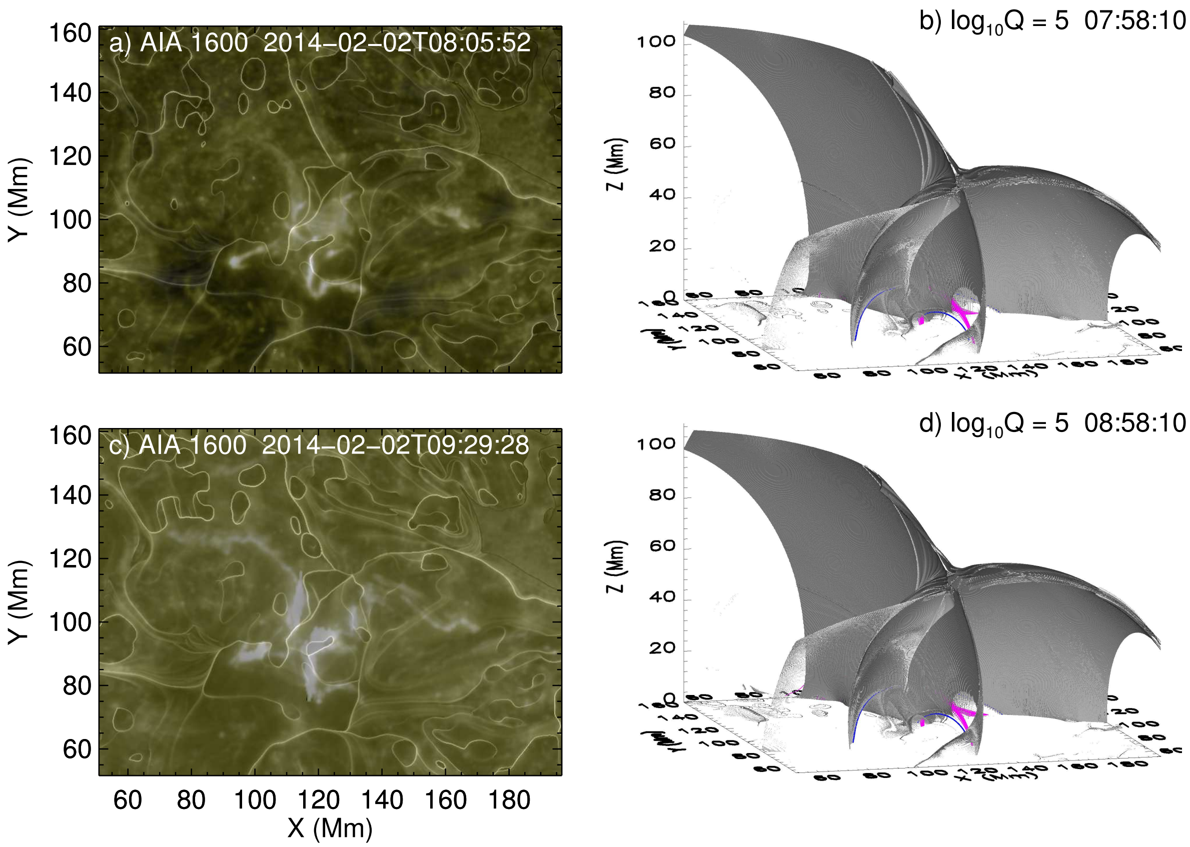

Supp. Fig. 6: Magnetic topology relevant to the M2.2 flare at 08:20 UT and M4.4 flare at 09:31 UT on 2014 February 2. Left column: A UV 1600 Å image taken during the impulsive phase of the flare and remapped with the CEA projection is blended with the $\log Q$ map calculated for the HMI magnetogram acquired at approximately the same time. Right column: Isosurfaces of $\log Q = 5$, overplotted by field lines traced in the neighborhood of the double null, with 'fan' lines in magenta and 'spine' lines in blue. A 360 deg side view of the $\log Q = 5$ isosurfaces in Panels (b) and (d) is provided in Supplementary Movies 8 and 9, respectively.

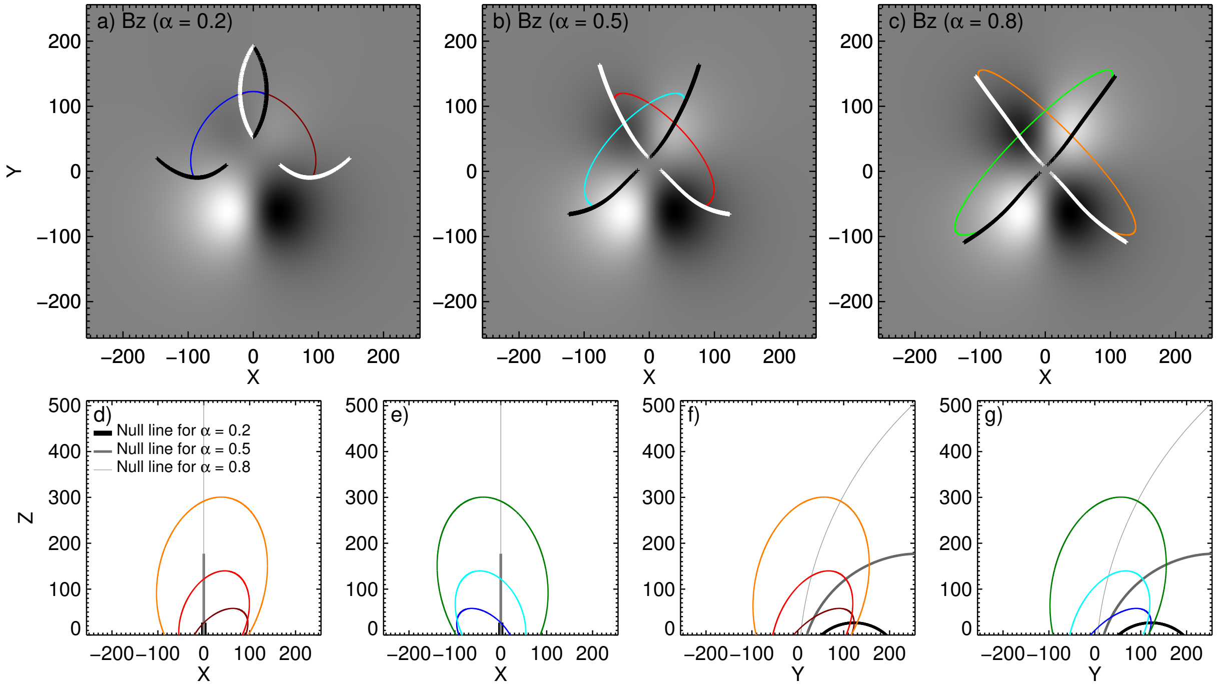

Supp. Fig. 7: Idealized quadrupole field. Panels (a–c) show the map of B_z at $z = 0$ with different α. The null lines for $\alpha = 0.2,\ 0.5,\ 0.8$ are shown in black, gray, and light gray colors, respectively, with decreasing thickness, in bottom panels. Exemplary spine field lines threading the middle of each null line are also projected in X-Y (a–c), X–Z (d, e), and Y–Z (f, g) planes. The spines resulting from tracing field lines toward the null are shown in maroon ($\alpha = 0.2$), red ($\alpha = 0.5$), and orange ($\alpha = 0.8$), whose footpoints are shown in white in top panels, while those resulting from tracing field lines away from the null are shown in blue ($\alpha = 0.2$), cyan ($\alpha = 0.5$), and green ($\alpha = 0.8$), whose footpoints are shown in black.

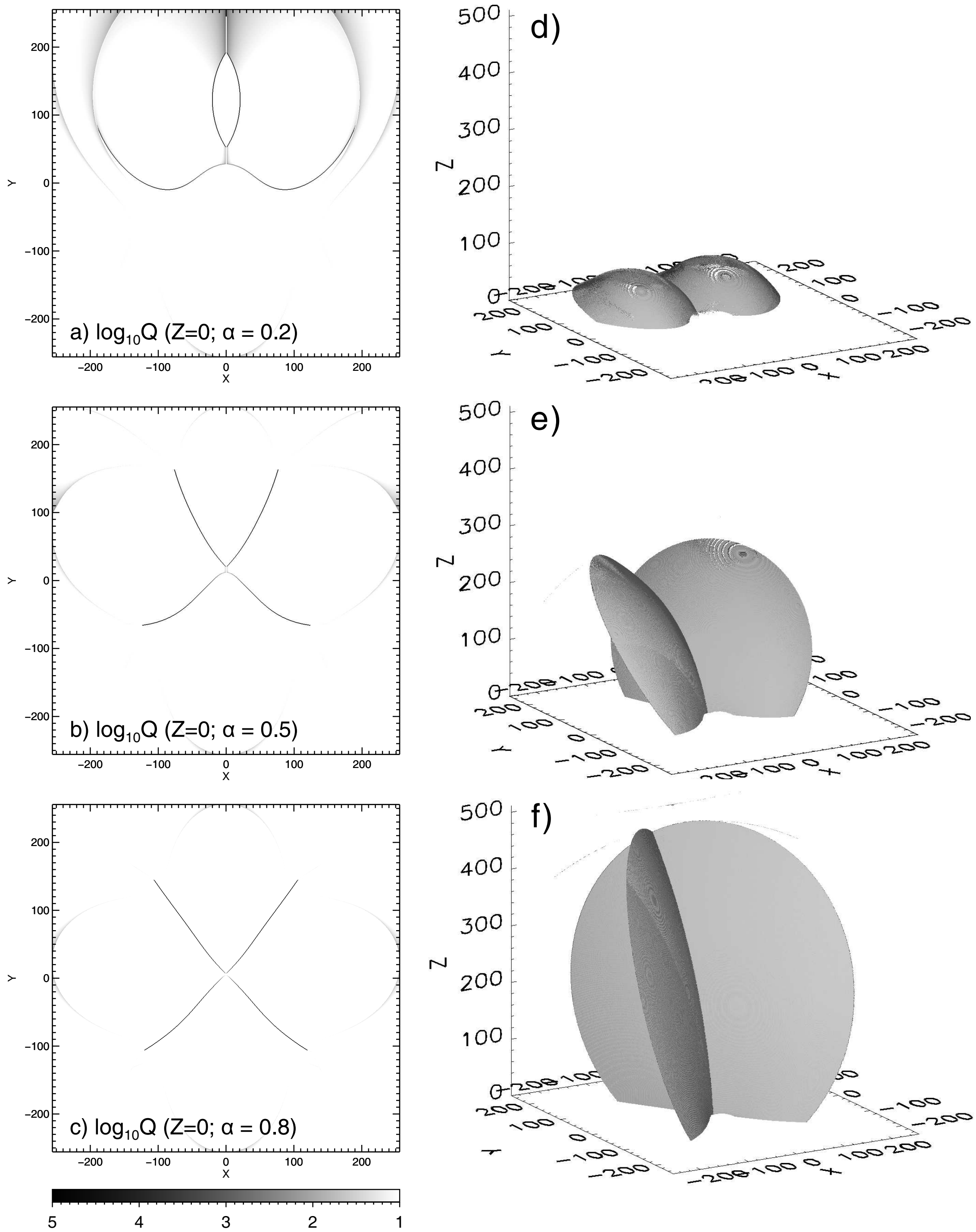

Supp. Fig. 8: Magnetic topology of the quadruple field with different α. The left column shows the maps of $\log_{10} Q$ at $z = 0$. The corresponding isosurfaces of $\log_{10} Q = 4$ in a 3D perspective are shown on the right column.

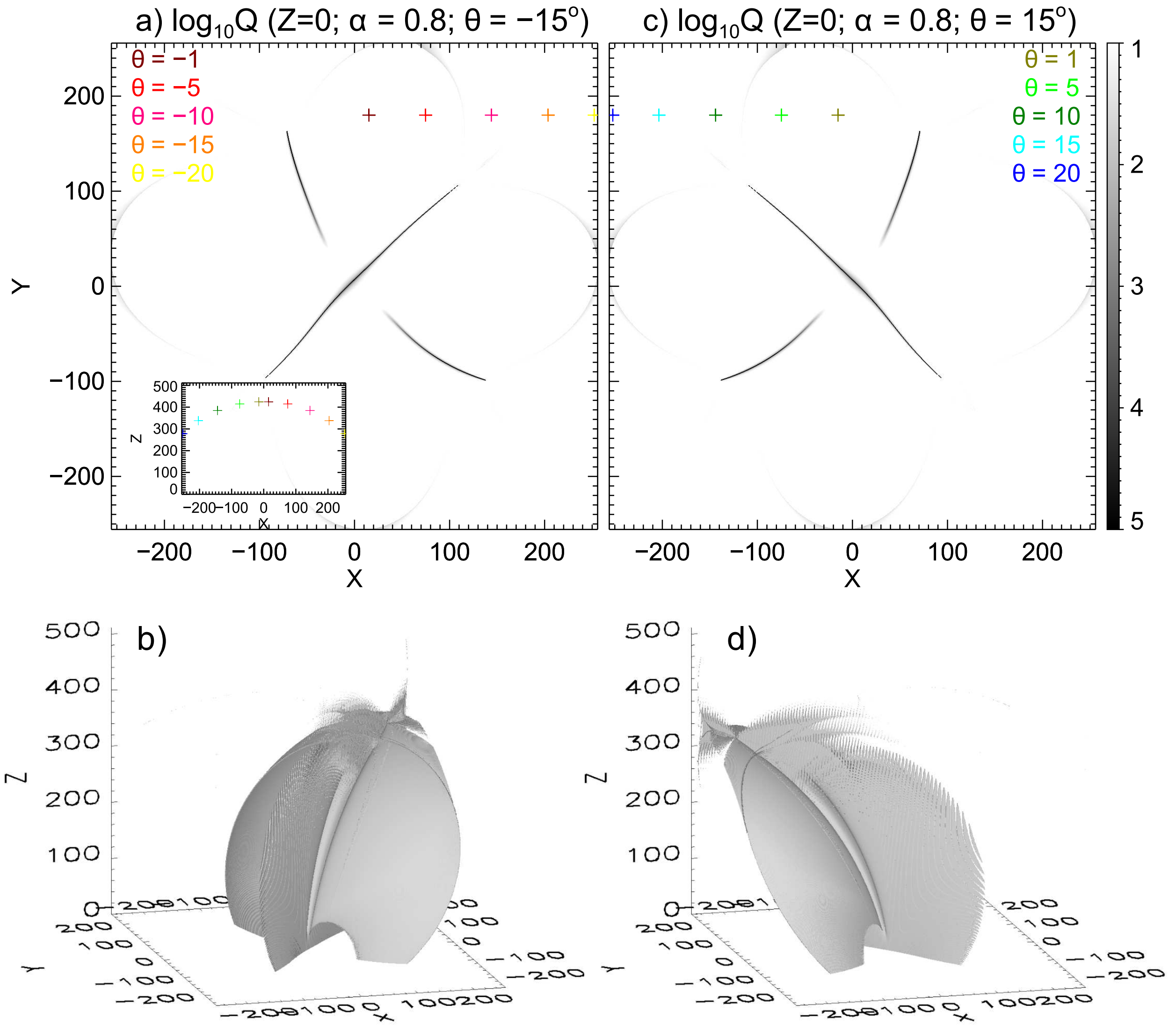

Supp. Fig. 9: Magnetic topology of the quadrupole field when $\mathbf{m}_2 = \alpha \times 1.024 \times 10^9 \times (\cos\theta,\, \sin\theta,\, 0)^T$. Top panels show the maps of $\log_{10} Q$ at $z = 0$ when $\theta = -15°$ (a) and $15°$ (c). The corresponding isosurfaces of $\log_{10} Q = 4$ are shown in (b) and (d), respectively. The crosses in the top panels mark the null locations with different θ values (color coded). The inset in (a) shows the null locations in the X-Z plane.

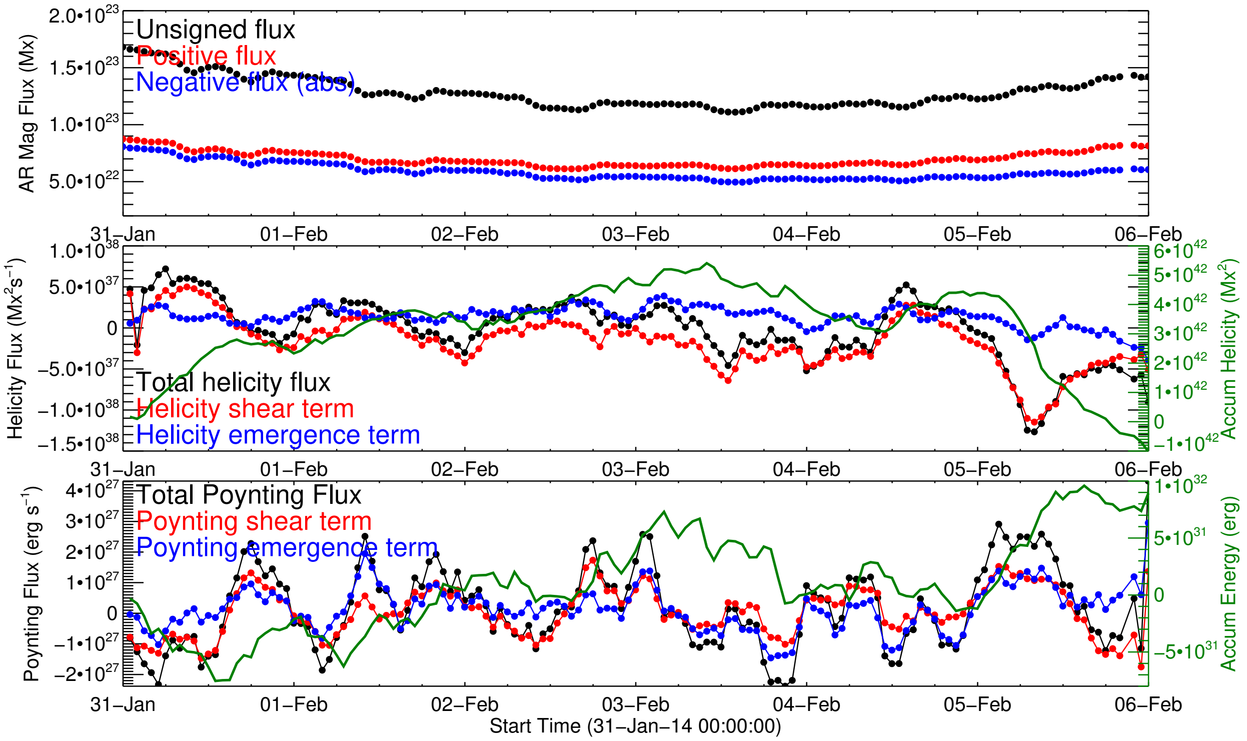

Supp. Fig. 10: Helicity and energy injection into AR 11967. From top to bottom we show the time series of magnetic fluxes [Mx], helicity fluxes [Mx^2 s^{-1}], and Poynting fluxes [erg s^{-1}]. The helicity [Mx^2] and magnetic energy [erg] accumulated in the active region are displayed in green in the corresponding panel, scaled by the right y-axis.

Supplementary Movies

Supplementary Movie 1 AIA observation of the X-shaped major flare (XMF) at 18:11 UT on 2014 February 2 (cf. Figure 1)

Supplementary Movie 2 Evolution of NOAA AR 11967 from January 31 to February 5 (Figure 3)

Supplementary Movie 3 $\log Q$ at different heights for the potential field at 17:58 UT on 2014 February 2 (cf. Figure 4(b–d))

Supplementary Movie 4 360 deg side view of the isosurfaces of $\log Q = 5$ for the potential field at 17:58 UT on 2014 February 2, superimposed by field lines traced in the neighbourhood of two nulls, with fan (spine) field lines in magenta (blue) (cf. Figure 5).

Supplementary Movie 5 $\log Q$ at different heights for the NLFFF at 17:58 UT on 2014 February 2 (cf. Figure 6(c and d))

Supplementary Movie 6 AIA observation of the XMF at 08:20 UT on 2014 February 2 (cf. Supplementary Figure 1)

Supplementary Movie 7 AIA observation of the XMF at 09:31 UT on 2014 February 2 (cf. Supplementary Figure 2)

Supplementary Movie 8 360 deg side view of the isosurfaces of $\log Q = 5$ for the potential field at 07:58 UT on 2014 February 2, superimposed by field lines traced in the neighbourhood of two nulls, with fan (spine) field lines in magenta (blue) (cf. Supplementary Figure 6(b)).

Supplementary Movie 9 360 deg side view of the isosurfaces of $\log Q = 5$ for the potential field at 08:58 UT on 2014 February 2, superimposed by field lines traced in the neighbourhood of two nulls, with fan (spine) field lines in magenta (blue) (cf. Supplementary Figure 6(d))
